# Monoclonal antibody ONS-M21 recognizes integrin α3 in gliomas and medulloblastomas

**DOI:** 10.1038/sj.bjc.6690053

**Published:** 1999-01

**Authors:** H Kishima, K Shimizu, K Tamura, Y Miyao, E Mabuchi, E Tominaga, J Matsuzaki, T Hayakawa

**Affiliations:** Department of Neurosurgery, Osaka University Medical School, 2-2 Yamadaoka, Osaka, 565-0871, Japan; Exploratory Research Laboratory Chugai Pharmaceutical Co. Ltd,1-135 Komakado, Gotenba, Shizuoka 412-8153, Japan

**Keywords:** glioma, medulloblastoma, monoclonal antibody, integrin α3

## Abstract

The monoclonal antibody ONS-M21 recognizes an antigen found on the surface of glioma and medulloblastoma cells but does not react with the antigens of normal brain tissue. We purified and identified the 140-kDa protein by means of an antibody-binding affinity column. This 140-kDa antigen has sequences homologous to the amino-terminal region and five parts of the internal domain of integrin α3. When the integrin α3-related sequences was amplified and used to analyse the mRNA of glioma and medulloblastoma surgical specimens, the transcription level of integrin α3 mRNA appeared to be quantitatively correlated with the grade of malignancy. These findings suggest that the ONS-M21 antibody, which reacts with integrin α3, might be useful in the diagnosis of gliomas and medulloblastomas. © 1999 Cancer Research Campaign


*British Journal of Cancer* (1999) **79**(2), 333–339
© 1999 Cancer Research Campaign

### Monoclonal antibody ONS-M21 recognizes integrin α3 in gliomas and medulloblastomas

**H Kishima[1], K Shimizu[1], K Tamura[1], Y Miyao[1], E Mabuchi[1], E Tominaga[2], J Matsuzaki[2] and T Hayakawa[1]**

[1]Department of Neurosurgery, Osaka University Medical School, 2-2 Yamadaoka, Suita, Osaka 565-0871, Japan, and [2]Exploratory Research Laboratory Chugai Pharmaceutical Co. Ltd, 1-135 Komakado, Gotenba, Shizuoka 412-8153, Japan

**Summary** The monoclonal antibody ONS-M21 recognizes an antigen found on the surface of glioma and medulloblastoma cells but does not react with the antigens of normal brain tissue. We purified and identified the 140-kDa protein by means of an antibody-binding affinity column. This 140-kDa antigen has sequences homologous to the amino-terminal region and five parts of the internal domain of integrin α3. When the integrin α3-related sequences was amplified and used to analyse the mRNA of glioma and medulloblastoma surgical specimens, the transcription level of integrin α3 mRNA appeared to be quantitatively correlated with the grade of malignancy. These findings suggest that the ONS-M21 antibody, which reacts with integrin α3, might be useful in the diagnosis of gliomas and medulloblastomas.

**Keywords:** glioma; medulloblastoma; monoclonal antibody; integrin α3

Medulloblastomas are the most common type of primitive neuroectodermal tumours observed in the central nervous system of children. In spite of recent advances in therapy, including microsurgery, combined chemotherapy and radiotherapy, the long-term prognosis for patients with these tumours is not satisfactory (Evans et al, 1990), pointing to a need for more specific and efficient therapies. Antibodies that recognize medulloblastoma cells are expected to act as diagnostic and therapeutic reagents. Therefore, we attempted to produce a medulloblastoma-specific monoclonal antibody (MAb). Immunization of mice with ONS-76 medulloblastoma cells (Tamura et al, 1989) successfully produced the antibodies referred to as ONS-M21 MAb (Moriuchi et al, 1993). ONS-M21 MAb reacted with a surface antigen of most glioma and medulloblastoma cell lines, and it also reacted with surgical specimens of gliomas and medulloblastomas. On the other hand, this MAb did not show cross-reactivity with peripheral blood cells or normal brain tissue. However, mouse antibodies are highly immunogenic in humans, and, hence, their clinical use may be limited. We genetically engineered a recombinant, humanized version of ONS-M21 MAb (hONS-M21 Ab) by means of CDR grafting (Ohtomo et al, 1995) in order to eliminate immunogenicity. This humanized Ab competed with ONS-M21 MAb for binding to glioma cells. In addition, this Ab, when labelled with an isotope, was capable of depicting tumour location by an autoradiographic technique. Thus, humanized Ab possesses various advantages that make it attractive for clinical applications.

As the target molecule of ONS-M21 remains unknown, identification of this antigen is a necessary step before any clinical use of this antibody can be attempted. In the present study, we purified ONS-M21 antigen from ONS-76 cell lysates by means of an Ab-binding affinity column, analysed the amino acid sequences of the ONS-M21 binding protein and identified the target molecule of ONS-M21 MAb. In addition, the mRNA level of this antigen was analysed by reverse transcription–polymerase chain reaction (RT-PCR) in surgical specimens of gliomas and medulloblastomas.

*Received 24 September 1997*
*Revised 29 April 1998*
*Accepted 30 April 1998*

*Correspondence to:* K Shimizu

### MATERIALS AND METHODS

### Cell culture conditions and preparation of the monoclonal antibody

The human medulloblastoma cell line ONS-76 was established in our laboratory (Tamura et al, 1989; Institution for fermentation, Osaka, No. 50355), maintained in Dulbecco's modified Eagle medium (DMEM) with 10% heat-inactivated fetal bovine serum (FBS) and 50 μg ml$^{-1}$ gentamycin, and incubated in a humidified 10% carbon dioxide-in-air atmosphere at 37°C. ONS-76 cells were collected using a cell scraper and washed with cold Tris-buffered saline (TBS; 10 mmol l$^{-1}$ Tris-Cl, 150 mmol l$^{-1}$ NaCl, pH 8.0). The cells were resuspended in a detergent solution (TBS with 0.5% Tween-20 and 1 mg ml$^{-1}$ *p*-amidinophenyl methane-sulphonyl fluoride hydrochloride) at a concentration of $10^6$ cells ml$^{-1}$, and then shaken for 2 h at 4°C to elute the cell extract. After centrifuging (3000 r.p.m., 20 min), the supernatant was collected as cell extract and stored at –80°C.

ONS-M21 MAb (IgG1) was purified as previously described (Moriuchi et al, 1993).

### IMMUNOPRECIPITATION

To label ONS-76-producing proteins with [$^{35}$S]methionine, ONS-76 cells were preincubated overnight in methionine-free MEM (ICN Biomedicals, Costa Mesa, CA, USA), and then cultured in methionine-free MEM with 10% FBS and 100 μCi ml$^{-1}$ of an L-[$^{35}$S] in vivo cell labelling mix (Amersham, Buckinghamshire, UK) for 5 h in a humidified 10% carbon dioxide-in-air atmosphere at 37°C. The cell extract was then obtained as described above. This cell extract (1 ml) was preincubated with 50 μl of protein A–Sepharose (Pharmacia, Uppsala, Sweden) conjugated with rabbit anti-mouse IgG1 (Cappel, Durham, NC, USA) for 1 h at

4°C. After centrifuging, the supernatant was incubated at 4°C with 80 μg of ONS-M21 MAb for 1 h and then with 50 μl of protein A–Sepharose conjugated with rabbit anti-mouse IgG1 for 1 h at 4°C. In order to compare this antibody with another commercially available antibody (P1B5; Dakopatts, Glostrup, Denmark) that recognizes integrin α3, the cell extract was preimmunoprecipitated with P1B5 Ab instead of ONS-M21 MAb, centrifuged and the supernatant reimmunoprecipitated with ONS-M21 MAb. The final immunoprecipitate was washed five times with TBS and then boiled in sample buffer containing 62.5 mM Tris-Cl (pH 6.8), 10% glycerol, 2% sodium dodecyl sulphate (SDS), 2% 2-mercaptoethanol and 0.1% bromophenol blue for 5 min to separate protein A–Sepharose and rabbit immunoglobulins. The precipitate was applied to a 6% SDS polyacrylamide gel electrophoresis (SDS-PAGE). The dried gel was autoradiographed by exposure to a BAS-IIIs imaging plate (Fuji Photo Film, Tokyo, Japan) for 12 h and analysed with BAS 1000 (Fuji Photo Film). The negative control was incubated with non-immune mouse IgG1 (Tago Company, Burlington, CA, USA) instead of ONS-M21.

### Purification of ONS-M21 antigen

To obtain a sufficient amount of the antigen, $1.2 \times 10^{10}$ cells were lysed as described above. The cell extract was dialysed to decrease the Tween-20 concentration to 0.1%. Cyanogen bromide (CNBr)-activated Sepharose (Pharmacia) was coupled with 20 mg of ONS-M21 monoclonal antibody and the coupled gel was embedded into a column (0.8 × 5 cm) and kept at 4°C. The cell extract was applied to the column at a rate of 5–10 ml $h^{-1}$. After washing the column with 1000 ml of TBS containing 0.1% Tween-20, bound proteins were eluted in 500-μl fractions with 0.05 M glycine, 0.1% Tween-20 and 0.5 M sodium chloride (pH 2.5) at a flow rate of 8 ml $h^{-1}$, and then immediately neutralized with 1 M Tris-Cl (pH 8.0). To estimate the concentration of the target protein, 10 μl of each fraction was blotted onto a nitrocellulose membrane and analysed by dot-blot enzyme-linked immunosorbent assay (ELISA) using 30 μg $ml^{-1}$ ONS-M21 as the primary antibody and 1:500 of peroxidase-conjugated goat anti-mouse IgG (Zymed, San Francisco, CA, USA) as the secondary antibody. Immunocomplexes were revealed using the ECL system (Amersham) and the absorbency was measured using the Image Quant program (Molecular Dynamics, Sunnyvale, CA, USA). Positive fractions were mixed together, dialysed in TBS containing 0.1% Tween-20, and reapplied to the affinity column. The bound protein was eluted and analysed as described above. Positive fractions were used for amino acid sequence analysis.

### Analysis of the amino acid sequence of the ONS-M21 antigen

The eluted sample was condensed 30-fold using a Centricon 50 concentrator (Amicon, Beverly, MA, USA). To analyse the amino-terminal amino acid sequence, 10% of the sample was separated by SDS-PAGE and electroblotted (1 mA $cm^{-2}$, 90 min) onto a PVDF membrane (Bio-Rad, Hercules, CA, USA). The membrane was stained with 0.1% Ponceau S and 2% acetic acid. The main band (140 kDa) was cut out and the N-terminal amino acid sequence of the isolated peptide was analysed on a protein sequencer (476A Protein Sequencer, Applied Biosystems). To digest the protein at methionine residues, the isolated band was dissolved in 70% formic acid containing 2 mg of CNBr and then incubated for 18 h at room temperature. The suspension was concentrated and dried by the spin-vacuum method. The digested peptide sample was resuspended in 0.1% trifluoroacetic acid and separated by microbore high-performance liquid chromatography (HPLC) on a Vydac C18 column (5 μm, 2.1 × 250 mm; Vydac, Hesperia, CA, USA) using an increasing concentration gradient of acetonitrile (0–90%, 0.2 ml $min^{-1}$, 0.9% $min^{-1}$). Each peak was analysed by the sequencer.

### Immunohistochemistry

Surgical specimens of gliomas and medulloblastomas were embedded in OCT compound (Tissue Tek, Miles, IN, USA) and immediately frozen. Immunohistochemical analysis was performed according to the previously described method (Moriuchi et al, 1993). In brief, specimens were fixed with acetone, blocked with gout normal serum, and incubated with primary Abs, such as ONS-M21 MAb, anti-Mac-1 Ab (PharMingen, SanDiego, CA, USA), or anti-glial fibrillary acid protein (GFAP) Ab (Dakopatts). Then, biotinylated goat anti-mouse or anti-rabbit IgG (Dakopatts) was added as secondary Ab, the samples were washed with PBS, incubated with streptavidin–biotin–peroxidase complex, and immunocomplexes were revealed by the peroxidase reaction using 0.06% diaminobenzidine with 0.01% hydrogen peroxidase in 50 mM Tris-HCl buffer (pH 7.0).

### Isolation of total RNA and reverse transcription

Surgical specimens were treated with Isogen (Nippongene, Tokyo, Japan) and the total RNA was extracted according to the protocol provided by the manufacturer. Total RNA (10 μg) was converted to cDNA using oligo-dT primer $pd(T)^{12-18}$ (0.5 μg, Invitrogene, San Diego, CA, USA) as a primer and avian myeloblastosis virus (AMV) reverse transcriptase (28 units, Seikagaku Kogyo, Tokyo, Japan) in 30 μl of reverse transcription buffer containing 50 mM Tris-HCl (pH 8.3), 100 mM potassium chloride, 10 mM magnesium chloride, and 600 μM of each dNTP (dATP, dCTP, dGTP and dTTP, Perkin Elmer Cetus, Norwalk, CT, USA), 10 mM dithiothreitol and 10 units of ribonuclease inhibitor (Takara, Kyoto, Japan). The reaction mixture was incubated at 42°C for 50 min, heated at 70°C for 10 min, chilled on ice and then stored at –20°C.

### Polymerase chain reaction of cDNA

On the basis of published sequences (Takada et al, 1991; Tsuji et al, 1991), we prepared pairs of oligonucleotide primers corresponding to sequences of the integrin α3 gene and the housekeeping gene glyceraldehyde-3-phosphate dehydrogenase (GAPDH) as a control. The following gene-specific oligo-nucleotide primers were used: integrin α3 (2154–2173) sense primer 5′-GCCAAGC-TAATGAGACCATC-3′, antisense primer (2742–2761) 5′-TCAG-CACGAGTGCTCGAGACTTG-3′, GAPDH (211–231) sense primer 5′-CCCATCACCATCTTCCAGGAG-3′, antisense primer (475–495) GTTGTCATGGATGACCTTGGC-3′. The amplified products of integrin α3 and GAPDH were 608 and 285 bp respectively.

One-thirtieth of the RT reaction mixture was used as a source of cDNA. Each polymerase chain reaction (PCR) (100 μl) contained 40 pmol of each primer, 2.5 units of *Taq* polymerase (Amplitaq Gold, Perkin Elmer Cetus), 160 μmol/$l^{-1}$ of each dNTP, 10 μl of

 © Cancer Research Campaign 1999

1 × PCR buffer, 2 mmol $l^{-1}$ magnesium chloride and cDNA. The reaction was performed in a PCR thermal cycler (Perkin Elmer Cetus). After preheating the mixture at 95°C for 9 min, the material for PCR was denatured at 94°C for 1 min, annealed at 55°C for 1 min and extended at 72°C for 2 min. Typically, 25–40 cycles of amplification were performed. PCR products were size fractionated by gel electrophoresis through 2% agarose. The density of the separated bands was evaluated using Image Quant (Molecular Dynamics).

### RESULTS

### Molecular weight of the ONS-M21 antigen

In order to determine the molecular weight of the ONS M-21 antigen, we performed immunoprecipitation of $^{35}$S-labelled cell extracts of ONS-76 with ONS-M21 MAb. Autoradiography of the sample revealed a 140-kDa band, which was not detected in the negative control lane (Figure 1, lanes 2 and 3). Other weak bands were seen in both lanes. We therefore verified that ONS-M21 MAb recognizes the 140-kDa protein.

### Separation of eluted fractions from the affinity column

Portions of the eluted fractions from the ONS-M21 mAb-fixed affinity column were blotted onto a nitrocellulose membrane and analysed by the dot-blot ECL system (Amersham). The absorbency of the blots was measured and fractions 10–16 were found to be positive (Figure 2A). A part of the collected sample was separated by SDS-PAGE and stained with silver. One major band migrating with a molecular weight of 140 kDa and one minor band of about 38 kDa were detected (Figure 2B).

### Amino acid sequence analysis reveals ONS-M21 antigen to be integrin α3

About 10% of the purified sample was concentrated and separated by SDS-PAGE. The main band (140 kDa) was then cut out and the amino acid sequence was analysed. Nineteen of the 20 amino acids from the amino terminus were identified (Figure 3). A homology search revealed that the 18-amino-acid sequence was identical to the amino-terminal sequence of human integrin α3 (Takada et al, 1991; Tsuji et al, 1991).

The cyanogen bromide-treated sample was applied to a HPLC column to separate the digested peptides. Although many peaks were detected, five peptides were chosen for amino acid sequence analysis. All of the identical sequences showed complete matches with the internal sequence of integrin α3 (Figure 3; Takada et al, 1991; Tsuji et al, 1991).

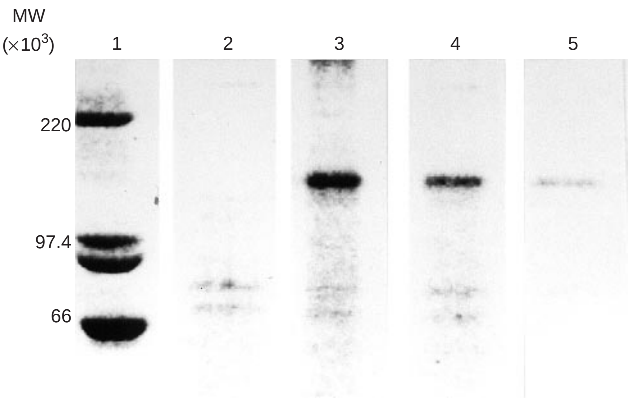

**Figure 1** Molecular weight of the ONS-M21 MAb-recognized antigen. Whole-cell extracts of ONS-76 that were labelled with [$^{35}$S]methionine were immunoprecipitated with ONS-M21 MAb or P1B5 Ab or control mouse IgG. The precipitates were then analysed using 6% SDS-polyacrylamide gels. Lane 1, molecular weight standards; molecular weights are indicated in kDa. Lane 2, ONS-76 extract incubated with control IgG. Lane 3, ONS-76 extract incubated with ONS-M21 MAb. Lane 4, ONS-76 extract incubated with P1B5 Ab. Lane 5, ONS-76 extract incubated with ONS-M21 MAb following immunoprecipitation with P1B5 Ab

### ONS-M21 MAb recognizes the same antigen as the commercially available anti-integrin α3 Ab

We performed immunoprecipitation with ONS-M21 MAb and commercially available anti-integrin α3 Ab, P1B5 Ab, from $^{35}$S-labelled cell extracts of ONS-76. Both Abs recognized a protein with a molecular weight of 140 kDa (Figure 1, lanes 3 and 4). The supernatant of the cell extract, which had been already immunoprecipitated with P1B5 Ab, was reimmunoprecipitated with ONS-M21 MAb. The 140-kDa band in lane 5 of Figure 1 was extremely faint compared with that of lane 3. This suggests that P1B5 Ab and ONS-M21 MAb interact with a common antigen.

### Protein expression and mRNA transcription of the ONS-M21 antigen gene in surgical specimens of gliomas and medulloblastomas

Surgical specimens of gliomas and medulloblastomas were classified according to the histological classification of tumours of the central nervous system (Daumas-Duport et al, 1988; Kleihues and Burger, 1993). In tumour specimens of grade II gliomas, scattered staining with ONS-M21 MAb was immunohistochemically observed (Figure 4A). The number of positive cells increased in grade III gliomas (data not shown). Many positive cells were detected in glioblastoma (grade IV) (Figure 4B). In surgical specimens, cells recognized by ONS-M21 MAb were not positively stained with anti-Mac-1 Ab and anti-GFAP Ab (Figure 4D and E). All things considered, these cells would appear not to be microglial cells or reactive astrocytes, but rather tumour cells.

As surgical specimens do not always suffice to purify a sufficient amount of RNA, RT-PCR had to be used instead of Northern blotting analysis. To determine the appropriate number of cycles for quantitative evaluation of the amount of cDNA, the correlation between the PCR cycle number and the amount of RT-PCR product was examined. Through preliminary evaluation, we chose 25 cycles of PCR for GAPDH cDNA and 35 cycles for integrin α3 cDNA. These RT-PCR products were mixed at a GAPDH to integrin α3 volume ratio of 0.5, and they were separated using 2% agarose gel electrophoresis (Figure 5A). Integrin α3 mRNA transcription expressed as a ratio of that of the housekeeping gene GAPDH is shown in Figure 5. There was a tendency for integrin α3 mRNA transcription to increase with the glioma grade of malignancy (Figure 5B).

### DISCUSSION

In this report, we have demonstrated that the target molecule of monoclonal antibody ONS-M21 is human integrin α3. The molecular weight of this antigen was previously reported to be 90-kDa

© Cancer Research Campaign 1999 

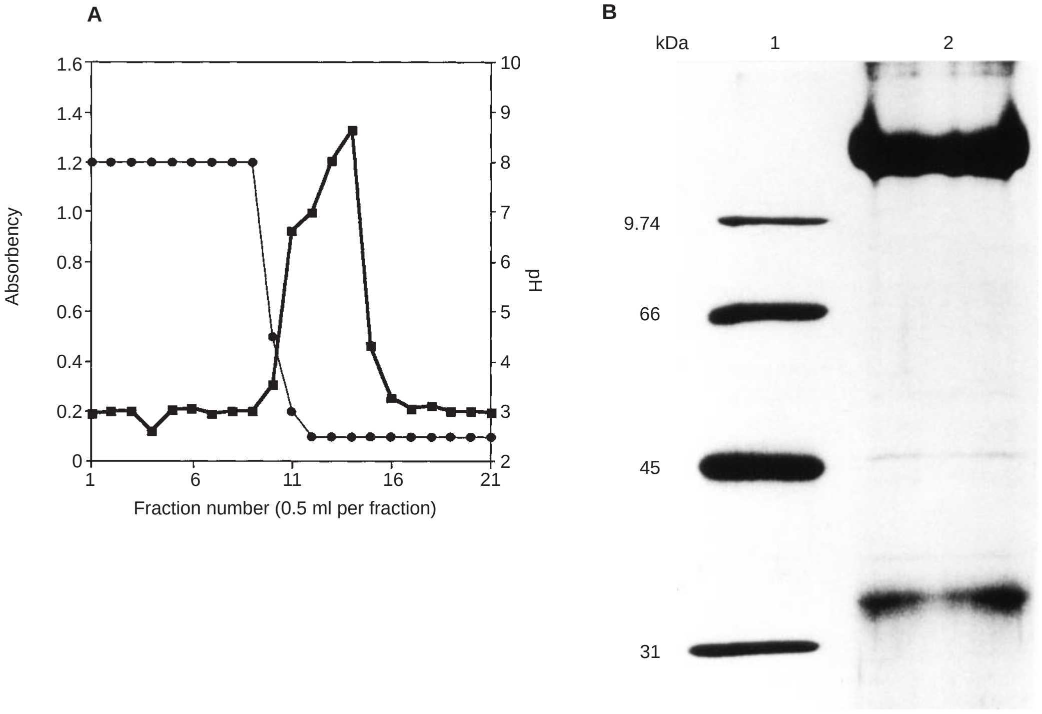

**Figure 2** Purification of ONS-M21 antigen. (**A**) The antigen recognized by ONS-M21 MAb from ONS-76 cell extracts was purified using ONS-M21 MAb-fixed affinity chromatography. The bound protein was eluted with acid buffer and the antigen concentration of each fraction was determined by immunoblotting using an ECL system. Squares represent the absorbency of dot blots. Circles represent the pH of the eluted samples. (**B**) SDS-PAGE of the ONS-M21 antigen purified using ONS-M21 MAb-affinity column chromatography. Positive samples of ECL dot blots were mixed together, condensed, separated by 7.5% SDS-PAGE and stained with silver. Lane 1, molecular weight standards; molecular weights are in kDa. Lane 2, eluted sample

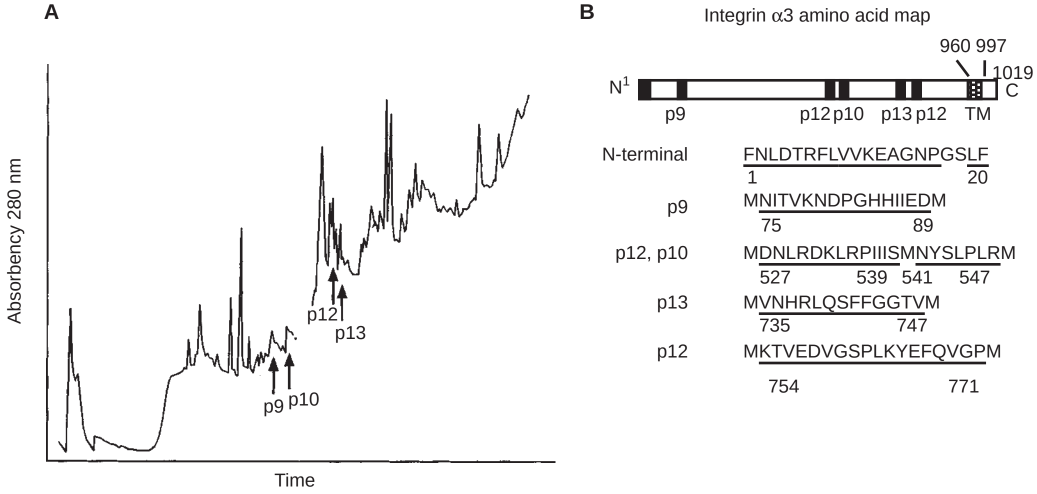

**Figure 3** Amino acid sequence of ONS-M21 antigen. (**A**) CNBr-digested peptide map of the protein eluted from the ONS-M21 affinity column. The bound protein was treated with 70% formic acid containing 2 mg of CNBr. The peptides were dissolved in 0.1% trifluoroacetic acid and analysed by HPLC. The amino acid sequences of p9, 10, 12 and 13 were obtained. (**B**) The sequences of the amino-terminal and internal domains were obtained from the purified antigen. The sequences were aligned with residues 1–20, 75–89, 527–539, 541–547, 735–747 and 754–771 of the deduced amino acid sequence of integrin α3. TM, transmembrane region of integrin α3; N, amino-terminal; C, carboxy-terminal protein (Moriuchi et al, 1993), which is at least 50 kDa smaller than the integrin α3 detected in this report. This discrepancy turns out to be due to an error of the molecular weight marker in the previous report. Hence, the exact molecular weight should now be considered to be 140 kDa, as described in this report (Figure 1). Two molecular species of protein were adsorbed to the ONS-M21 MAb-binding column (Figure 2). Sequence analysis revealed the protein of the major band to be integrin α3. This analysis also demonstrated that the other 38-kDa protein had homology with the P32 subunit of pre-mRNA splicing factor SF2 (Honore et al, 1993). However, no

© Cancer Research Campaign 1999

A

B

C

D

E

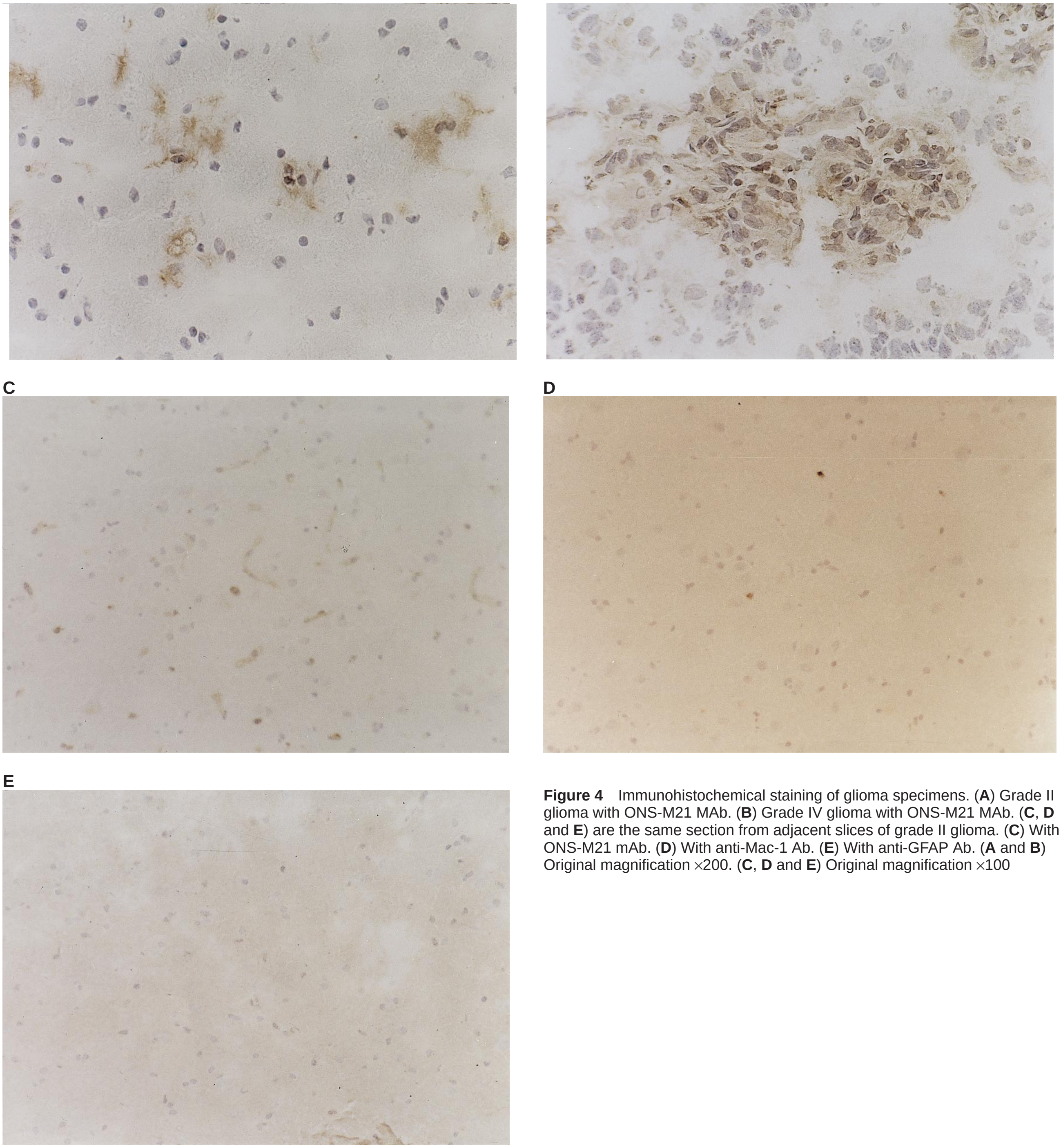

Figure 4 Immunohistochemical staining of glioma specimens. (**A**) Grade II glioma with ONS-M21 MAb. (**B**) Grade IV glioma with ONS-M21 MAb. (**C**, **D** and **E**) are the same section from adjacent slices of grade II glioma. (**C**) With ONS-M21 mAb. (**D**) With anti-Mac-1 Ab. (**E**) With anti-GFAP Ab. (**A** and **B**) Original magnification ×200. (**C**, **D** and **E**) Original magnification ×100

positive band other than the 140-kDa band was detected by immunoprecipitation. We also purified this antigen from the subcutaneously transplanted ONS-76 cells of nude mice. SDS-PAGE and silver staining revealed the presence of the 140-kDa band but no 38-kDa band was detected (data not shown). Commercially available P1B5 Ab, which recognized integrin α3, could precipitate the same molecular weight protein as ONS-M21 MAb (Figure 1). In the competitive immunoprecipitation experiment, prior treatment of the samples with P1B5 Ab resulted in failure of ONS-M21 MAb to precipitate the antigen, suggesting that P1B5 Ab and ONS-M21 MAb compete for the same antigen (Figure 1). These results, taken together, suggest that the ONS-M21 antigen is identical to integrin α3.

According to the literature, integrin α3 is not distributed on oligodendrocytes, ependymal cells, neurons or microglias (Paulus et al, 1993). Our experiment showed that cells recognized by ONS-M21 MAb are neither microglias nor reactive astrocytes (Figure 4). This also supports the idea that ONS-M21 MAb reacts with integrin α3 expressed on glioma cells.

Furthermore, to investigate for mutations of this protein expressed in glioma cells, we examined the cDNA sequence of the

© Cancer Research Campaign 1999 

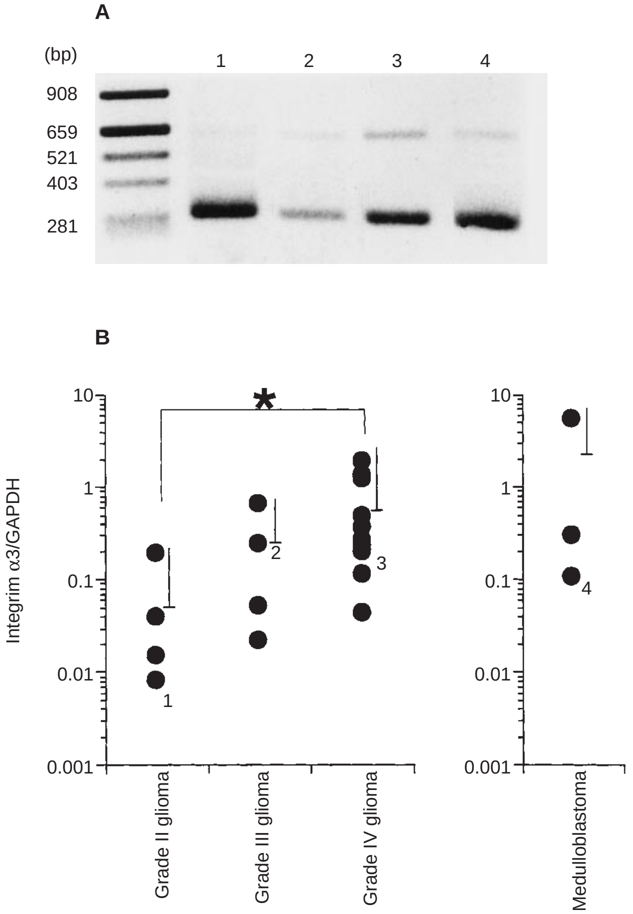

**Figure 5** Correlation between the glioma grade of malignancy and the mRNA transcription level of integrin α3. (**A**) Electrophoretic pattern of RT-PCR products from surgical specimens. Lane 1, surgical specimen of glioma grade II. Lane 2, surgical specimen of glioma grade III. Lane 3, surgical specimen of glioma grade IV. Lane 4, surgical specimen of medulloblastoma. Upper band (608 bp) and lower band (285 bp) indicate the amplified PCR products of integrin α3 and GAPDH respectively. (**B**) Transcription of integrin α3 mRNA in surgical specimens of gliomas and medulloblastomas. RT-PCR products were separated by electrophoresis and their absorbency was measured using a densitometer. Integrin α3 mRNA expression was evaluated as the ratio of absorbency of the amplified band of integrin α3 to that of GAPDH. Numbers in the figure correspond to the lane numbers in (**A**). Horizontal bars represent the mean value of the ratio of the absorbencies. Vertical bars indicate standard errors. There is a statistical difference between grade II glioma and grade IV glioma. Statistical evaluation by Student's *t*-test. $^*P < 0.01$

coding region of integrin α3 in five glioma cell lines. No common cDNA mutations causing amino acid replacements or deletions were detected among these cell lines (data not shown). These findings suggest that ONS-M21 MAb recognizes the normal form of integrin α3 expressed in these glioma cell lines.

Many monoclonal antibodies against gliomas, prepared by immunizing mice with glioma cells, have been reported. Some of those antibodies have been analysed with regard to their target molecules (Schold et al, 1993; Okada et al, 1994; Romejin et al, 1994). Mouse MAbs against gliomas have also been applied in experimental models. Some Abs were expected to have an anti-tumour effect by themselves (Dastider and Sharma, 1995), and others were used in combination with a radioisotope (Williams et al, 1990) or chemotherapeutic agent (Schrappe et al, 1992). Furthermore, some MAbs have also been applied to clinical diagnosis and therapies, even though these Abs were derived from mice (Daghighian et al, 1993; Schold et al, 1993; Faillot et al, 1996). Human Ab is preferred for clinical applications because mouse Ab can be more immunogenic than human Ab. Although human MAbs against gliomas have been produced, their target molecules have not been reported (Dan et al, 1992, 1995). Recombinant humanized ONS-M21 Ab has already been successfully produced (Ohtomo et al, 1995), and therefore hONS-M21 Ab is the only Ab against gliomas whose target molecule has been identified. The expression of integrin α3 in the normal brain has been reported to be very low or even undetectable (Fradet et al, 1984; McGeer et al, 1990; Paulus et al, 1993), and ONS-M21 MAb is not reactive to either fetal or adult brain tissues (Moriuchi et al, 1993). A significant amount of ONS-M21 MAb attached to the cell surface was rapidly internalized into the cytoplasm, and the A-chain of ricin-conjugated ONS-M21 MAb displayed glioma specific cell toxicity in vitro (Shimizu et al, 1998). $^{125}$I-labelled hONS-M21 Ab was found to accumulate in the glioma of nude rat brains 24 h after intravenous administration (data not shown). In this way, hONS-M21 Ab is attractive for clinical applications such as for pathological diagnosis or glioma-specific targeting.

Integrin α3 is one of the adherent molecules that binds to collagen, laminin and fibronectin (Elices et al, 1991; Takeda et al, 1991), although it is still controversial as to whether integrin α3 induces cell–cell adhesion (Shiramarao et al, 1993; Weitzmen et al, 1995). The expression of integrin α3 in fibroblasts increases following oncogenic transformation (Tsuji et al, 1991). In malignant tumours, poorly differentiated hepatocellular carcinomas show down-regulation of integrin α3 (Jaskiewicz and Chasen, 1995), an abundant expression of which is related to the mode of gastric and colorectal cancer invasion (Boku et al, 1995). Furthermore, transfection with cDNA coding for integrin α3 reduced the tumorgenicity of rhabdomyosarcoma cells (Weitzman et al, 1996). Thus, the expression of integrin α3 is different depending on the type of cancer and the level of malignancy. Although immunohistochemical analysis of integrin α3 in gliomas has been demonstrated (Paulus et al, 1993), no quantitative evaluation has been reported. In the present study, we quantified integrin α3 mRNA by an RT-PCR method using GAPDH genes as an internal control, in accordance with previous reports (Suzuki et al, 1995; Zhao et al, 1995). The results showed that integrin α3 was transcribed in all gliomas and medulloblastomas and that the amount of integrin α3 increased with the grade of the malignancy. The results of this study suggest that integrin α3 may play some role during the development of malignancy in glioma, such as invasion or infiltration of the surrounding brain. It would be interesting to investigate the pathophysiological mechanisms of the ONS-M21 antigen, which we have shown in this report to be integrin α3.

### ACKNOWLEDGEMENT

This work was supported by a grant-in-aid for scientific research (08457363) from the Ministry of Education, Science, Sports and Culture of Japan.

### REFERENCES

Boku N, Yoshida S, Ohtsu A, Fujii T, Koba I, Oda Y, Ryu M, Matsumoto T, Hasebe T, Hosokawa K, Yama T, Saito D, Moriya N and Abe K (1995) Expression of integrin α3 in gastric and colorectal cancers: Its relation to wall construction and mode of invasion. *Jpn J Cancer Res* **86**: 934–940

 © Cancer Research Campaign 1999

Daghighian F, Pentlow KS, Larson SM, Graham MC, DiResta GR, Yeh SDJ, Macapinlac H, Finn RD, Arbit E and Cheung NKV (1993) Development of a method to measure kinetics of radiolabelled monoclonal antibody in human tumor with applications to microdosimetry: Positron emission tomography studies of iodine-124 labeled 3F8 monoclonal antibody in glioma. *Eur J Nucl Med* **20**: 402–409

Dan MD, Schlachta CM, Guy J, McKenzie RG, Dorsscheid DR, Sandor VA, Villemure JG and Price GB (1992) Human antiglioma monoclonal antibodies from patients with astrocytic tumors. *J Neurosurg* **76**: 660–669

Dan MD, Earley EM, Griffin MC, Maiti PK, Prashar AK, Yuan XY, Friesen AD and Kaplan HA (1995) Human monoclonal antibody BT32/A6 and a cell cycle-independent glioma-associated surface antigen. *J Neurosurg* **82**: 475–480

Dastidar SG and Sharma SK (1995) Monoclonal antibody against human glioblastoma multiforme (U-87MG) immunoprecipitates a protein of molecular weight 38 kDa and inhibits tumor growth in nude mice. *J Neuroimmunol* **56**: 91–98

Daumas-Duport C, Scheithauer B, O'Fallon J and Kelly P (1988) Grading of astrocytoma. A simple and reproducible method. *Cancer* **62**: 2152–2165

Elices MJ, Urry LA and Hemler ME (1991) Receptor function for the integrin VLA-3: Fibronectin, collagen, and laminin binding are differentially influenced by ARG-GLY-ASP peptide and by divalent cations. *J Cell Biol* **112**: 168–181

Evans AE, Jenkin RD, Sposo R, Ortega JA, Wilson CB, Wara W, Ertel IJ, Kramer S, Chang CH, Leikin SL and Hammond GD (1990) The treatment of medulloblastoma. Results of a prospective randomized trial of radiation therapy with and without CCNU, vincristine, and predonisone. *J Neurosurg* **72**: 572–582

Faillot T, Magdelent H, Mady E, Stasiecki P, Fohanno D, Gropp P, Poisson M and Delattre JY (1996) A phase I study of an anti-epidermal growth factor receptor monoclonal antibody for the treatment of malignant gliomas. *Neurosurgery* **39**: 478–483

Fradet Y, Cardo CC, Thomson T, Daly ME, Whitmore WF Jr, Lloyd KO, Melamed MR and Old LJ (1984) Cell surface antigen of human bladder cancer defined by mouse monoclonal antibody. *Proc Natl Acad Sci USA* **81**: 224–228

Honore B, Madsen P, Rasmussen HH, Vandekerckhove J, Celis JE and Leffers H (1993) Cloning and expression of a cDNA covering the complete coding region of the P32 subunit of human pre-mRNA splicing factor SF2. *Gene* **134**: 283–287

Jaskiewicz K and Chasen MR (1995) Differential expression of transforming growth factor alpha, adhesion molecules and integrin in primary, metastatic liver tumors and in cirrhosis. *Anticancer Res* **15**: 559–562

Kleihues P and Burger PC (1993) *Histological Typing of Tumors of the Central Nervous System*. Springer: Berlin

McGeer PL, Zhu SG and Dedhar S (1990) Immunostaining of human brain capillaries to antibodies to very late antigens. *J Neuroimmunol* **26**: 213–218

Moriuchi S, Shimizu K, Miyao Y and Hayakawa T (1993) Characterization of a new mouse monoclonal antibody (ONS-M21) reactive with both medulloblastomas and gliomas. *Br J Cancer* **68**: 831–837

Ohtomo T, Tsuchiya M, Sayo K, Shimizu K, Moriuchi S, Miyao Y, Akimoto T, Akamatsu K, Hayakawa T and Ohsugi Y (1995) Humanization of mouse ONS-M21 antibody with the aid of hybrid variable regions. *Mol Immunol* **32**: 406–407

Okada H, Yoshida J, Seo H, Wakabayashi T, Sugita K and Hagiwara M (1994) Anti-(glioma surface antigen) monoclonal antibody G-22 recognizes overexpressed CD44 in glioma cells. *Cancer Immunol Immunother* **39**: 313–317

Paulus W, Baur I, Schuppan D and Roggendorf W (1993) Characterization of integrin receptors in normal and neoplastic human brain. *Am J Pathol* **143**: 154–163

Romeijin P, Lenthall R, Stavrou D, Melcher D, Ladyman H and Ritter MA (1994) Identification of glioma-associated antigen MUC 2–63 as CD44. *Br J Cancer* **70**: 799–803

Schold SC, Zalutsky MR, Coleman RE, Glantz MJ, Friedman AH, Jaszczak RJ, Bigner SH and Bigner DD (1993) Distribution and dosimetry of I-123-labeled monoclonal antibody 81C6 in patients with anaplastic glioma. *Invest Radiol* **28**: 488–496

Schrappe M, Bumol TF, Apelgren LD, Briggs SL, Koppel GA, Markowittz DD, Mueller BM and Reisfeld RA (1992) Long-term growth suppression of human glioma xenografts by chemoimmunoconjugates of 4-desacetylvinblastine-3-carboxyhydrazide and monoclonal antibody 9.2.27. *Cancer Res* **52**: 3838–3844

Shimizu K, Park KC, Tamura K, Kishima H, Kawata H, Yoshimura Y, Sekimori Y, Miyao Y and Hayakawa T (1998) Internalization with high targeting potential of mouse monoclonal antibody ONS-M21 recognizing human malignant glioma antigen. *Cancer Lett* **27**: 171–176

Shiramarao P, Steffner P and Gehlsen KR (1993) Biochemical evidence for a homophilic interaction of the α3β1 integrin. *J Biol Chem* **268**: 22036–22041

Suzuki H, Shibata H, Murakami M, Sato A, Naito M, Ichihara A, Matsumoto A, Tsujimoto G, Hiarasawa A, Horie K and Saruta T (1995) Modulation of angiotensin II type 1 receptor mRNA expression in human blood cells: Comparison of platelets and mononuclear leucocytes. *Endo J* **42**: 15–22

Takada Y, Murphy E, Pil P, Chen C, Ginsberg MH and Hemler ME (1991) Molecular cloning and expression of the cDNA for α3 subunit of human α3β1 (VLA-3), an integrin receptor for fibronectin, laminin, and collagen. *J Cell Biol* **115**: 257–266

Tamura K, Shimizu K, Yamada M, Okamoto Y, Matsui Y, Park KC, Mabuchi E, Moriuchi S and Mogami H (1989) Expression of major histocompatibility complex on human medulloblastoma cells with neuronal differentiation. *Cancer Res* **49**: 5380–5384

Tsuji T, Hakomori SI and Osawa T (1991) Identification of human galactoprotein b3, an oncogenic transformation-induced membrane glycoprotein, as VLA-3 α subunit: the primary structure of human integrin α3. *J Biochem* **109**: 659–665

Weitzman JB, Chen A and Hemler ME (1995) Investigation of the role of integrins in cell–cell adhesion. *J Cell Sci* **108**: 3635–3644

Weitzman JB, Hemler ME and Brodt P (1996) Reduction of tumorigenicity by α3 integrin in a rhabdomyosarcoma cell line. *Cell Adh Comm* **4**: 41–52

Williams JA, Wessels BW, Edwards JA, Kopher KA, Wanek PW, Whram MD, Order SE and Klein JL (1990) Targeting and therapy of human glioma xenografts *in vivo* utilizing radiolabeled antibodies. *Cancer Res* **50**: 974–979

Zhao J, Nobuhito A and Nishimoto SK (1995) Quantitation of matrix Gla protein mRNA by competitive polymerase chain reaction using glyceraldehyde-3-phosphate dehydrogenase as an internal control. *Gene* **155**: 159–165

© Cancer Research Campaign 1999
